# SARS-CoV-2 in semen: a multicenter prospective study and literature review

**DOI:** 10.1186/s12610-024-00236-z

**Published:** 2024-12-02

**Authors:** Giovanni Cochetti, Michele Del Zingaro, Giuseppe Maiolino, Alessio Paladini, Matteo Mearini, Riccardo Schiavina, Lorenzo Bianchi, Lorenzo De Angelis, Mattia Panciarola, Barbara Camilloni, Eugenio Brunocilla, Ettore Mearini

**Affiliations:** 1 Department of Medicine and Surgery, Urology Clinic, University of Perugia, Perugia, Italy; 2 Urology Department, Santa Maria Hospital, Terni, Italy; 3 Department of Urology, University of Bologna, St. Orsola-Malpighi Hospital, Bologna, Italy; 4 Microbiology and Clinical Microbiology Section, Department of Medicine and Surgery, University of Perugia, Perugia, Italy

**Keywords:** Semen, COVID-19, Polymerase Chain Reaction, Infertility, Erectile dysfunction, Sperme, COVID-19, Réaction en Chaîne par Polymérase, Infertilité, Dysfonction érectile

## Abstract

**Background:**

Despite numerous efforts to demonstrate the presence of the SARS-CoV-2 in semen of affected males, no clear evidence exists. We conducted a multicenter prospective study on adult patients with a confirmed diagnosis of SARS-CoV-2 including patients with active infection (Active Group) and with a history of COVID-19 disease at least of 6 months (Recovered Group). An RT-PCR test for SARS-CoV-2 and a semen analysis were performed on the semen of the enrolled patients. Genital/sexual symptoms were investigated in both groups. In the active infection group, urinary and sexual functions were assessed in the active phase and after 6 months. Finally, the literature on the detection of SARS-CoV-2 in semen was reviewed non-systematically.

**Results:**

Sixty-five patients were enrolled (Active Group = 15, Recovered Group = 50). RT-PCR testing for SARS-CoV-2 found no trace of the virus in any of the semen samples. Genital/sexual symptoms during the active phase were reported in 8 (12.2%) patients. No statistically significant differences in semen quality were found between the two groups. IPSS and IIEF-5 scores did not change significantly during the different phases of infection about (*p* > 0.05).

**Conclusions:**

SARS-CoV-2 was not detected in semen of acute or recovered cases. Sperm parameters were not significantly different in the two groups. Urinary and erectile functions appeared stable across the phases of infection.

**Supplementary Information:**

The online version contains supplementary material available at 10.1186/s12610-024-00236-z.

## Résumé

**Contexte:**

Malgré de nombreux efforts pour démontrer la présence du SRAS-CoV-2 dans le sperme des hommes concernés, il n’existe aucune preuve claire. Nous avons mené une étude prospective multicentrique chez des patients adultes ayant un diagnostic confirmé de SRAS-CoV-2, comprenant des patients atteints d’une infection active (groupe actif) et des patients ayant un antécédent de COVID-19 depuis au moins 6 mois (groupe guéri). Un test RT-PCR pour le SARS-CoV-2 et une analyse de sperme ont été effectués sur le sperme des patients inclus. Les symptômes génitaux/sexuels ont été étudiés dans les deux groupes. Dans le groupe d’infection active, les fonctions urinaires et sexuelles ont été évaluées dans la phase active et après 6 mois. Enfin, la littérature sur la détection du SRAS-CoV-2 dans le sperme a été examinée de manière non systématique.

**Résultats:**

Soixante-cinq patients ont été recrutés (groupe actif = 15, groupe guéri = 50). Les tests RT-PCR pour le SRAS-CoV-2 n’ont trouvé aucune trace du virus dans les échantillons de sperme. Des symptômes génitaux/sexuels pendant la phase active ont été rapportés chez 8 patients (12,2 %). Aucune différence statistiquement significative dans la qualité du sperme n’a été observée entre les deux groupes. Les scores de l’International Prostate Symptom Score (IPSS) et de L’ Index International    de la Fonction Erectile (IIEF-5) n’ont pas changé de manière significative au cours des différentes phases de l’infection.

**Conclusions:**

Le SRAS-CoV-2 n’a pas été détecté dans le sperme des cas aigus ou guéris. Les paramètres spermatiques n’étaient pas significativement différents entre les deux groupes. Les fonctions urinaires et érectiles étaient stables tout au long des phases de l’infection.

## Background

The coronavirus disease 2019 (COVID-19) outbreak resulted in unforeseen health, societal, and economic repercussions. Starting from January 2021, different aspects and evidence about a relationship between the infection and uro-andrological issues emerged, ranging from the management of resources and their impact on therapeutical and treatment algorithms [1–3] to the biological impact of the infection on uro-andrological diseases [4, 5] and the impact on psychological health of urological patients [6].

While various viruses, including the mumps, Zika, Ebola, Marburg, Hepatitis B, Hepatitis C, Human Immunodeficiency, Human papillomavirus and Herpes, are able to infect male genitals and enter human semen[7, 8], the potential for severe acute respiratory syndrome coronavirus 2 (SARS-CoV-2) to infect the male genital system remains a subject of controversy. The detection of SARS-CoV-2 in human semen could have several implications, including the possibility of a new potential transmission route for the infection, the potential to alter testicular function, and ultimately an impact on Assisted Reproductive Technologies.

Semen is a complex fluid comprising spermatozoa and other products originating from the testes, combined with secretions from the accessory sex glands, including the epididymides, prostate, seminal vesicles, and bulbourethral glands [9], so SARS-CoV-2 in semen could arise from various sources, including these organs. Despite numerous efforts to demonstrate the presence of the SARS-CoV-2 virus in the semen of affected males, clear evidence remains elusive nearly four years into the COVID-19 pandemic. Many studies suffer from limitations such as small sample sizes, and importantly substantial variations in sample types (age, disease severity, timing of sampling from infection onset, etc.), collection methods (risk of contamination, sample processing, etc.), and analytical techniques (polymerase chain reaction type, target genes, cut-offs used, etc.) [4, 10, 11]. In 2022, a systematic review and meta-analysis indicated that SARS-CoV-2 mRNA could be identified in semen with a higher probability during the acute phase of COVID-19 infection [4]. Several factors define the “acute phase”. While many infected patients clear the virus within a few weeks of infection, some individuals, particularly young adults, may experience a persistent long-term infection [12]. In such cases, a prolonged period between the acute and recovered phases is necessary to have the highest probability of true viral clearance. Consequently, the primary aim of our study was to assess the presence of the SARS-CoV-2 virus in the semen of patients with active infection (defined as a positive nasopharyngeal swab within the previous three days) and in recovered patients (where viral clearance is established by two consecutive negative nasopharyngeal swabs for at least six months). Secondary aims included comparing semen quality of the two groups and analyzing changes in urinary and erectile function scores during the acute and recovering phases of the disease.

## Methods

We conducted a multicenter prospective study involving adult patients with a confirmed diagnosis of SARS-CoV-2. The inclusion criteria comprised patients aged 18 – 60 years with active (Active Group) infection (a positive reverse transcription-polymerase chain reaction (RT-PCR) nasopharyngeal swab in the previous three days) and asymptomatic or mildly symptomatic COVID-19 disease, defined as respiratory symptoms without evidence of pneumonia or hypoxia according to WHO classification [13] and patients with a history of COVID-19 disease (of any severity grade) who had recovered from the infection (Recovered Group) as indicated by two consecutive negative RT-PCR nasopharyngeal swabs, for at least six months. For patients with active infection, we excluded severely symptomatic or hospitalized patients owing to the low probability of their producing a semen sample. Exclusion criteria for the study encompassed age < 18 or ≥ 60 years, indwelling urinary catheter, urinary tract infection, hormonal therapy or drugs impacting the hypothalamic–pituitary–gonadal axis, alpha-blocker therapy, anejaculation, retrograde ejaculation, or a confirmed diagnosis of couple infertility. To identify potential adult male participants, a preliminary screening involved examining the COVID-19 patient database of centers enrolled without any exclusion criteria during June 2021 and September 2022. From these datasets, 1000 adult male patients aged between 18 and 60 years were randomly selected. Candidates were contacted and screened for inclusion/exclusion criteria. Informed oral consent was obtained through recorded telephone interviews for patients who accepted to participate in the study. Patient data collected included age, BMI, smoking, alcohol consumption, Age-Adjusted Charlson Comorbidity Index, fatherhood (number of children), therapy for COVID-19 disease, urological or andrological conditions, urological or non-urological surgeries, symptoms related to COVID-19 (including genitourinary). Moreover, we collected data on the vaccination status of patients. Patients were instructed on the semen collection procedure and invited to visit the microbiology laboratories involved in the study. Timing from first nasopharyngeal diagnostic swab to semen sampling (days) was recorded. Data confidentiality was maintained through serial numbering, with limited access granted solely to the researchers. Data collection, analysis, and results elaboration were recorded on a designated computer with restricted access and double password protection (account and file access), known exclusively to the study researchers. The study has been complied with all the relevant national regulations, institutional policies and in accordance with the principles of Helsinki Declaration (2013) and has been approved by the authors' institutional review board.

Finally, a nonsystematic literature review of studies on the detection rate through PCR of SARS-CoV-2 in semen samples of infected patients was performed by using PUBMED and the following search strategy: “semen’’ OR ‘‘seminal fluid” OR “sperm” AND ‘‘COVID-19” OR “SARS-CoV-2”. All types of study were included, excepting for abstracts, guidelines, study protocols and meeting reports. No geographic restrictions were applied. Only English-language articles were included. The principal aim of the review was to evaluate the pool crude detection rate of SARS-CoV-2 in semen samples.

### Semen sampling, processing and PCR analysis

All participants were instructed to obtain a semen sample through masturbation without lubricant. We provided guidelines to minimize the risk of sample contamination: the collection should occur at least two hours after the last urination, followed by a meticulous hand and penis washing using soap. Subsequently, hands and the penis were dried, and, avoiding touching any surface, the semen was ejaculated into a sterile, wide-mouthed noncytotoxic container. The collected samples and analysis were performed in the laboratories of the participating centers. A sexual abstinence period of at least two days and a maximum of seven days was required, except for patients in the Active Group, where the abstinence period was not limited in order to obtain a sample within three days of the positive nasopharyngeal swab Semen samples were processed within one hour of ejaculation for analysis.

A volume of 300 μL from the semen sample was utilized for viral RNA extraction using the Microlab Nimbus IVD system (Seegene Inc, Seoul, South Korea) and amplified with Allplex™ SARS-CoV-2 assay (Seegene) targeting envelope (E), nucleocapsid (N), and the RNA-dependent-RNA-polymerase (RdRP) genes. The remaining portion of the sample was allowed to undergo liquefaction at 37 °C for 60 min, enabling subsequent sperm evaluation in accordance with the WHO Manual for the Laboratory Examination and Processing of Human Semen 6th edition [14]: total sperm count (10^6^ per ejaculate), sperm concentration (10^6^/ml), progressive motility (%) and normal forms (%) were collected.

Urinary and sexual function were assessed through self-administered International Prostate Symptom Score (IPSS) and International Index of Erectile Function 5-items (IIEF-5) questionnaires sent via email to patients in the Active Group. The initial evaluation took place during the acute infection, and a subsequent assessment was conducted six months after viral clearance.

### Statistical analysis

A descriptive statistical analysis was performed: numerical parametric variables are shown as mean ± standard deviation, numerical nonparametric variables as median (interquartile range) and categorical as n, percentage. T-test, Pearson's chi-squared and Mann–Whitney U tests were used to evaluate significant differences between Active Group vs Recovered Group patients at baseline. We evaluated statistically significant differences between the two groups in semen quality by the Mann–Whitney U-test. Finally, significant changes in IPSS and IIEF-5 scores during the active and recovery period of patients in the Active Group were evaluated by a Wilcoxon signed-rank test was performed. All reported *p* values are two-sided and statistical significance was set at 0.05. Statistical analysis was conducted using SPSS version 11.5 (SPSS, Chicago, Illinois, USA).

## Results

**Fig. 1:**
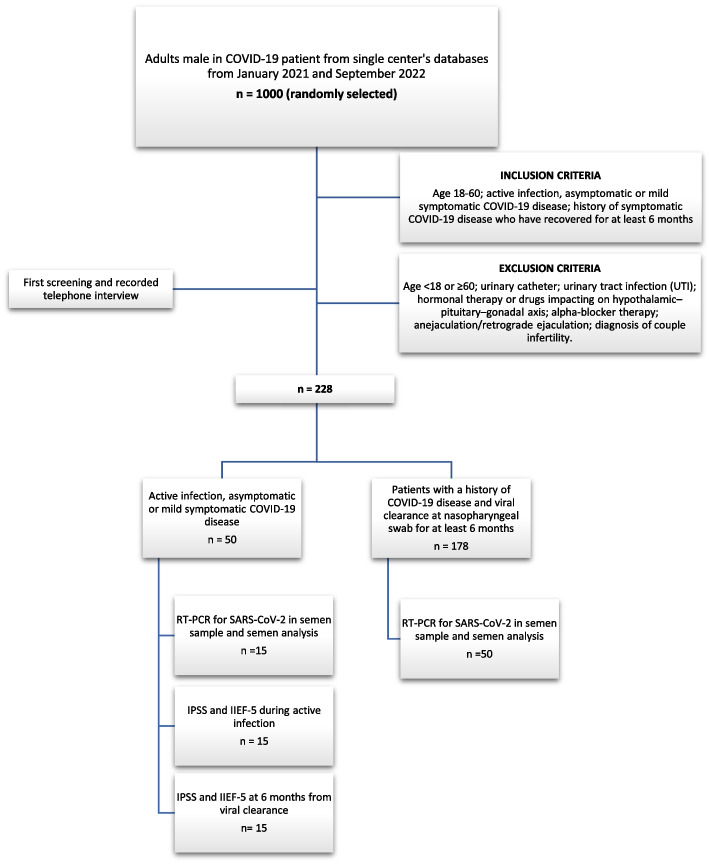
Flow diagram of study protocol and patient’s enrollment. Flow diagram of the study protocol, enrollment, and analysis. Screening eligibility (based on inclusion and exclusion criteria) and enrollment (acceptance to provide a semen sample) of our study. A randomly selected group of 1000 males aged between 18 and 60 years were selected from a COVID-19 database of the involved centers. After initial phone interview, 228 patients were eligible for the study but only 65 patients agreed to participate, and semen samples were obtained from 15 of 50 patients in the Active Group and from 50 of 178 patients in the Recovered Group (acceptance rate 36.5%, 65/178)

From the COVID-19 patient’s database of the two centers involved, we randomly selected 1000 males aged between 18 and 60 years. After initial phone interview with a screening based on inclusion and exclusion criteria, 228 patients were eligible for the study: 50 had asymptomatic or mildly symptomatic acute SARS-CoV-2 infection with positive nasopharyngeal swab in the previous three days (Active Group) and 178 had been negative for at least six months (Recovered Group). Only 65 patients agreed to participate in the study and semen samples were obtained from 15 of 50 patients in the Active Group and from 50 of 178 patients in the Recovered Group (acceptance rate 36.5%, 65/178). A flow diagram of study protocol is shown in Fig. 1.

**Fig. 2:**
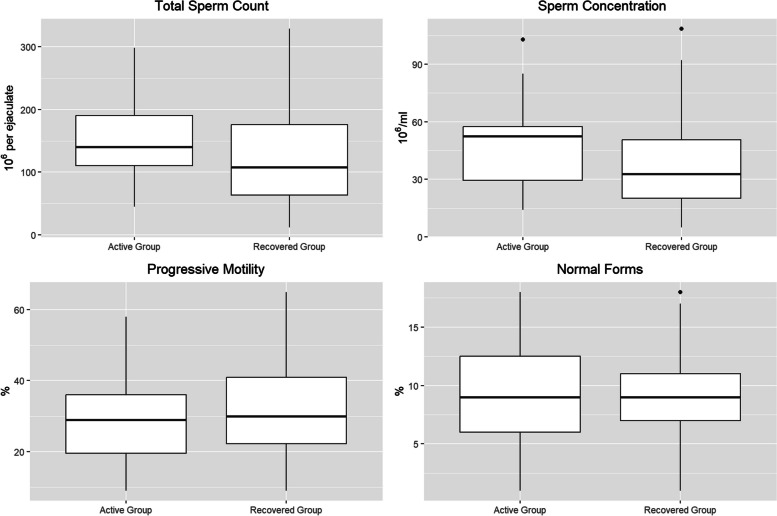
Sperm quality values in the Active Group (*n* = 15) and Recovered Group (*n* = 50). Box plots of semen quality parameters of the two groups: patients with active infection (Active Group, *n* = 15) vs patients with a history of COVID-19 disease at least of 6 months (Recovered Group, *n* = 50). No statistically significant differences were found in total sperm count (10^6^ per ejaculate), sperm concentration (10.^6^/ml), progressive motility (%) or normal forms (%)

Baseline, medical and COVID-19 disease data are shown in Table 1. In the total sample (*n* = 65), mean ± SD age and BMI was 34.7 ± 11.1 years and 26.5 ± 3.1 kg/m^2^ respectively. Only 5 (7.6%) and 22 (30.8%) of patients had a history of current urological and andrological disease, respectively. Median number of children was 1. Regarding “COVID-19 disease severity” and “timing from first nasopharyngeal diagnostic swab to semen sampling” a significant difference was found between the two groups. Genital or sexual symptoms arising during the active period of infection were reported in eight (12.2%) patients: three reported testicular pain, one a pain during erection, three inguinal-perineal discomfort and one a reduction in ejaculate volume. All enrolled patients were vaccinated against SARS-CoV-2: 50 subjects had been vaccinated twice and 15 subjects had received the vaccine booster.RT-PCR of SARS-CoV-2 did not reveal the presence of the virus in any of the samples analyzed, neither in the Active (*n* = 15) nor Recovered Group (*n* = 50). Table 2 and Fig. 2 show the values of semen quality of the two groups. No statistically significant differences were found in total sperm count (10^6^ per ejaculate), sperm concentration (10^6^/ml), progressive motility (%) or normal forms (%). Moreover, in the Recovered Group, 10 (20%) patients confirmed a pregnancy of their partner in the six months after infection.

**Table 1:** Demographic, clinical, and COVID-19-related characteristics of the Active (*n* = 15) and Recovered (*n* = 50) groups

		**Total Sample** (*n***= 65**)	**Active Group** **(*n* = 15)**	**Recovered Group (*n* = 50)**	***p***
Age (y), mean ± SD		34.7 ± 11.1	35.6 ± 10.7	34.4 ± 11.3	0.71
BMI (kg/m^2^), mean ± SD		26.5 ± 3.1	26.5 ± 2.6	26.5 ± 3.2	0.98
Smoking	Never	34 (52.3%)	9 (60%)	25 (50%)	0.72
	Former	23 (35.4%)	4 (26.7%)	19 (38%)	
	Current	8 (12.3%)	2 (13.3%)	6 (12%)	
Alcohol consumption	Never	13 (20%)	4 (26.7%)	9 (18%)	0.76
	Occasional (< 2 times/week)	47 (72.3%)	10 (66.7%)	37 (74%)	
	Frequent (> 2 times/week)	5 (7.7%)	1 (6.7%)	4 (8%)	
	Abuse (> 5 times/week)	0 (0%)	0 (0%)	0 (0%)	
Age-Adjusted Charlson Comorbidity Index, median (IQR)		0 (0 – 1)	0 (0 – 1)	0 (0 -1)	0.79
Fatherhood (number of children), median (IQR)		1 (0 – 2)	1 (0 – 2)	1 (0 -2)	0.82
Urological disease		5 (7.6%)	1 (6.7%)	4 (8%)	0.86
Andrological disease		20 (30.8%)	5 (33.3%)	15 (30%)	0.80
Uro-andrological surgeries		7 (10.8%)	2 (13.3%)	5 (10%)	0.71
COVID-19 severity	Asymptomatic	16 (24.6%)	6 (40%)	10 (20%)	**0.04**
	Mild	34 (52.3%)	9 (60%)	25 (50%)	
	Moderate	7 (10.8%)	0 (0%)	7 (14%)	
	Severe	5 (7.7%)	0 (0%	5 (10%)	
	Critical	3 (4.6%)	0 (0%)	3 (6%)	
Genital-sexual symptoms during SARS-CoV-2 infection		8 (12.2%)	3 (20%)	5 (10%)	0.30
Timing from first nasopharyngeal diagnostic swab to semen sampling (days), median (IQR)		197 (185.5 – 204.5)	2 (1 – 3)	200 (192.7 – 221)	** < 0.001**

**Table 2:** Comparison of RT-PCR results for SARS-CoV-2 and sperm analysis parameters (median, IQR) between the Active Group (*n* = 15) and the Recovery Group (*n* = 50)

	**Total Sample (*n* = 65)**	**Active Group (*n* = 15)**	**Recovered Group (*n* = 50)**	***p***
RT-PCR for SARS-CoV-2	0 (0%)	0 (0%)	0 (0%)	-
Total sperm count (10^6^ per ejaculate)	120.4 (63.7 – 179.5)	140 (100.4 -210.1)	107.5 (61.5 – 177.6)	0.20
Sperm concentration (10^6^/ml)	55.9 (38.4 – 78.5)	52.5 (25.1 – 58.4)	32.7 (18.9 – 51.2)	0.11
Progressive motility (%)	41 (29 – 49.2)	29 (17 – 41)	30 (21.7 – 41.2)	0.59
Normal forms (%)	12 (9 – 12)	9 (5 – 13)	9 (6.75 – 11)	0.68

Table 2 presents the RT-PCR results for detecting SARS-CoV-2 in the semen of the enrolled patients, along with a comparison of sperm quality between the Active Group and the Recovered Group. There were no statistically significant differences between the groups in terms of total sperm count, sperm concentration, progressive motility, or normal forms (Mann–Whitney U-test)

*RT-*PCR Reverse Transcription-Polymerase Chain Reaction, *SARS-CoV-2* Acute Respiratory Syndrome Coronavirus 2

In the Active Group (*n* = 15), median IPSS and IIEF-5 score during the infection were 5 (2 – 8.5) and 22 (20.5 – 25), respectively. After six months from recovery, median IPSS and IIEF-5 score were in 5 (2 – 9) and 22 (21 – 25), respectively, with no significant differences in the two scores from the period of active infection (*p* > 0.05). No patients started therapies for LUTS or erectile dysfunction after SARS-CoV-2 infection.

In Table 3 the results of the non-systematic review of literature are shown, including the present study. Among 827 patients analyzed in 29 studies with low-medium quality of evidence, 40.1% (332/827) were in the acute phase and 59.8% (495/827) in the recovered phase, although the definitions of disease phase widely varied in the reports. Only a small proportion had a contemporary (1 – 3 days) positive nasopharyngeal swab at the time of semen analysis. Most patients experimented a mild severity of COVID-19. The pooled crude detection rate was 1.6% (13/827). Only five studies found semen samples positive for SARS-CoV-2.

**Table 3:** Studies investigating the detection of SARS-CoV-2 in sperm within the medical literature

**Studies**	**City, country**	**Study type (Quality rating)**	**No. of men**	**Age **Mean ± SD or median (IQR) or median [range]	**% positive nasopharyngeal swab at time**^**#**^** of semen analysis**	**Clinical phase at time of semen analysis (AP, RP)**^**a**^	**Days from diagnosis**^**b**^Mean ± SD or median (IQR) or median [range]	**Severity of COVID-19**^**c**^	**Detection of SARS‐CoV‐2 in semen samples**	**Control**	**Orchitis-like symptoms**	**Semen parameters**
Song et al. 2020 [15]	Nanjing, China	Cohort (4)	12	31 [22–38]	1 (8.3%)	100% (12/12) RP	29.4 [14–42]	8.3% (1/12) A 91.7% (11/12) M	0% (0)	No	NM	NA
Ning et al. 2020 [16]	Wuhan, China	Cohort (4)	17	35 [23–46]	9 (52.9%)	100% (17/17) RP	27 [12–64]	47.0% (8/17) M 53.0% (9/17) S	0% (0)	No	2.7% (3/112) orchidoptosis	NA
Pan et al. 2020 [17]	Wuhan, China	Cross-sectional (4)	34	37 [18–55]	NR	100% (34/34) RP	31 [8–75]	100% (34/34) M/Mo	0% (0)	No	17.6% (6/34) orchitis-like symptoms	NA
Paoli et al. 2020 [18]	Italy	Case report (5)	1	31	1 (100%)	100% (1/1) RP	8	100% (1/1) M	0% (0)	No	NM	NA
Nicastri et al. 2020 [19]	Italy	Case report (5)	1	NR	NR	100% (1/1) AP	NR	100% (1/1) M	0% (0)	No	NM	NA
Li D et al. 2020 [20]	Shangqiu, China	Cohort (4)	38	NR	NR	39.5% (15/38) AP 60.5% (23/38) RP	NR	NR	15.8% (6/38) (4 AP, 2 RP)	No	NM	NA
Holtmann et al. 2020 [21]	Düsseldorf, Germany	Prospective Cohort control (3b)	20	42.2 ± 9.9 (for RP)	2 (16.6%)	100% (2/20) AP 100% (18/20) RP	45.2 [8–54] (for RP)	77.8% (14/18) M 22.2% (4/18) Mo	0% (0)	0% (*n* = 14)	1 (5.5%) testicular discomfort	Only Mo severity showed an impairment of sperm quality
Guo et al. 2021 [22]	Shandong, China	Case Series (4)	23	41.04 ± 11.56	0%	100% (23/23) RP	32 [26–34]	78.3% (18/23) M 21.7% (5/23) Mo	0% (0)	No	NM	100% (21/21) sperm counts, total motile sperm counts, and sperm morphology were normal
Ma et al. 2021 [23]	Wuhan, China	Cross-sectional (4)	12	31.5 [25–46]	1 (8.3%)	100% (12/12) RP	78.5 [56–109]	8.3% (1/12) M 91.7% (11/12) Mo	0% (0)	No	NM	66.7% (8/12) normal semen quality
Rawlings et al. 2020 [24]	San Diego, USA	Cross-sectional (4)	6	38 (mean)	6 (100%)	100% (6/6) AP	12 [6–17]	100% (6/6) M	0% (0)	No	NM	NA
Pavone et al. 2020 [25]	Palermo, Italy	Cross-sectional (4)	9	42 [28–60]	NR	22.2% (2/9) AP 77.8% (7/9) RP	39 [7–88]	11.1% (1/9) A 88.9% (8/9) M	0% (0)	No	NM	NA
Kayaaslan et al. 2020 [26]	Ankara, Turkey	Cross-sectional (4)	16	33.5 [18–54]	6 (37.5%)	100% (16/16) AP	1 (0–7)	68.8% (11/16) M 31.2% (5/16) Mo	0% (0)	No	NM	NA
Li H et al. 2020 [27]	Wuhan, China	Cross-sectional cohort study (4)	23	40.8 ± 8.5	23 (100%)	100% (23/23) AP	25.8 (mean)	60.9% (14/23) M 39.1% (9/23) Mo	0% (0)	0% (*n* = 22)	NM	39.1% (9/23) oligozoospermic 60.9% (14/23) significant increase in leucocytes
Ruan et al. 2021 [28]	Wuhan, China	Cross-sectional (4)	70^**d**^	30.5 [21–49]	0% (0)	100% (70/70) RP	NR	14.9% (11/74) M 41.9% (31/74) 43.2% (32/74) S	0% (0)	No	1.35% (1/74) scrotal discomfort (orchitis was ruled out by MRI)	Compared with healthy-control, sperm concentration, total sperm count and total motility were significantly declined (*n* = 55)
Temiz et al. 2021 [29]	Istanbul, Turkey	Cross-sectional (4)	20	NR	NR	50% (10/20) AP 50% (10/20) RP	NR	NR	0% (0)	No	NM	Sperm morphology was significantly lower in the COVID‐19 patients after treatment vs control (*n* = 10)
Best et al. 2021 [30]	Miami, USA	Prospective Cohort study (3)	16	NR	NR	100% (16/16) RP	NR	NR	0% (0)	No	3.4% (1/30) bilateral testis pain suggestive of orchitis	Concentration and total sperm number was significantly lower (*n* = 30) than control (*n* = 30)
Machado et al. 2021 [31]	Arkansas, USA	Cross-sectional study (4)	15	23 [19–43]	NR	100% (15/15) AP	4 [2–8]	13.3% (2/15) A 86.7% (13/15) M/Mo	6.6% (1/15)	No	NM	NA
Gacci et al. 2021 [32]	Italy	Prospective cross-sectional study (4)	43	[30 – 64]	0 (0%)	100% (43/43) RP	NR	12 (27.9%) NH 26 (60.5%) H 5 (11.6%) ICU	2.3% (1/43)	No	NM	25.6% (11/43) oligo-crypto-azoospermic
Paoli D et al. 2021 [33]	Italy	Prospective cross-sectional (3)	4	58.5 [28—61]	50% (2/4)	50% (2/4) AP 50% (2/4) RP	42.5 [17 – 61]	NR	0 (0%)	0	NM	Azoospermia (25%, 1/4) and asthenozoospermia (25%, 1/4)
Burke et al. 2021 [34]	Florida, USA	Cross-sectional (4)	19	32 [24–57]	52.6% (10/19)	57.9% (11/19) AP 42.1% (8/19) RP	6 [1–28]	5.3% (1/19) A 10.5% (2/19) M 84.2% (16/19) Mo	0% (0)	No	NM	NA
Gupta et al. 2021 [35]	New Delhi, India	Cross-sectional (4)	37	32.2 ± 5.6	NA	100% (37/37) AP	4.5 ± 0.5	64.9% (24/37) M 35.1% (13/37) A	0% (0)	No	NM	17/17 normal semen parameters in acute phase
Delaroche et al. 2021 [36]	France	Cross-sectional (4)	32	38.8 ± 10.9	100% (32/32)	100% (32/32) AP	4 [0—8]	16% (5/32) A 84% (27/32) Mo	3.1% (1/32)	No	0 (0%)	NA
Saylam et al. 2021 [37]	Turkey	Prospective cohort (2b)	30	35.7 ± 6.8	100% (30/30)	100% (30/30) AP	1	NR	13.3% (4/30)^**e**^	No	NM	NA
Sharma et al. 2021 [38]	India	Prospective observational study (4)	11	30 [24–40]	0 (%)	100% (11/11) RP	44 [19–59]	81.8% (9/11) M 18.2% ((2/11) Mo	0% (0)	No	0 (0%)	NA
Fraietta et al. 2022 [39]	Brazil	Prospective cohort (2b)	22	29 [23–33]	0%	100% (22/22) AP	6 [5–8]	91.0% (20/22) M 4.5% (1/22) Mo 4.5% (1/22) S	0%	No	9.1% (2/22)	No significant difference in seminal parameters at 7, 14 and 21 after the diagnosis (*n* = 14)
Donders et al. 2022 [40]	Belgium	Prospective observational study (3)	120^**f**^	34.7 ± 9.1	NR	100% (120/120) RP	52.7 ± 35.1	95.8% (115/120) NH 4.2% (5/120) H	0% (0)	No	NM	24.6% (29/118) normal 25.4% (30/118) oligozoospermic; 44.1% (52/118) asthenozoospermic 67.0% (79/118) teratozoospermic
Pavone C. et al. 2022 [41]	Italy	Cross-sectional (4)	36	41 (mean)	NR	50% (18/36) AP 50% (18/36) RP	15.0 [2.0–88.0]	8.3% (3/36) A 58.3% (21/36) M 33.3% (12/36) S	0 (0%)	No	NM	NA
Edimiris et al. 2023 [42]	Germany	Prospective case–control study (3)	25^**g**^ for three consecutive times	34.9 (mean)	25 (100%)	25 (100%) AP	4.4 (mean) 17.9 (mean) 81.7 (mean)	25 (100%) M	0% (0)	0% (0) (*n* = 12)	1 (4%) testicular pain	Semen parameter values did not differ significantly between subjects with mild COVID-19 and the control group (*n* = 12)
Present study	Italy	Prospective observational multicentre (3)	65	34.7 ± 11.1	23.1% (15/65)	23.1% (15/65) AP 76.9% (50/65) RP	197 (185.5 – 204.5)	24.6% (16/65) A 52.3% (34/65) M 10.8% (7/65) Mo 12.3% (8/65) S	0 (0%)	No	12.2% (8/65) genital-sexual symptoms	No significative difference in sperm parameters between active group and recovered group
**Studies *n* = 29**			**827**	**33.5** **Range: 18–64** **(*n* = 23)**	**-**	**40.1% (332/827) AP** **59.8% (495/827) RP**	**-**	**-**	**1.6% (13/827)**	**-**	**-**	**-**

This table presents findings from a nonsystematic literature review on the detection rate of SARS-CoV-2 in semen samples from infected patients, aiming to evaluate the overall crude detection rate. The data includes the studies referenced, their locations (city and country), study types with quality ratings, the number and age of male participants, and the percentage of positive nasopharyngeal swabs at the time of semen analysis. It also notes the clinical phase during semen analysis (acute or recovery), the duration from diagnosis, the severity of COVID-19 in participants, and the detection status of SARS-CoV-2 in semen samples. Additionally, the table reports on any control groups, the presence of orchitis-like symptoms, and the semen quality analyzed

*SD* Standard Deviation, *IQR* Interquartile range, *AP* Acute Phase, *RP* Recovering phase, *A* Asymptomatic, *M* Mild, *Mo* Moderate, *S* Severe, *H* Hospitalized, *NH* Non hospitalized, *ICU* Hospitalized with intensive care unit, *NM* Not Mentioned, *NA* Not Analyzed, ^#^Last 1–3 days

^a^Different criteria have been used to define “acute” and “recovery” phase (i.e. two continuous negative SARS-CoV-2 real-time reverse transcriptase-polymerase chain reaction (RT-PCR) assay of pharyngeal swab specimens or substantial resolution on chest CT scans with much lessened symptoms). We report “acute phase” (AP) and “recovery phase” (RP) according to definitions of each study

^b^Different criteria have been used to define the initial diagnosis (i.e. day when the symptoms were noticed or first positive pharyngeal swab or using anti-2019-nCoV antibodies)

^c^For patients in “recovery phase” severity of COVID-19 has been reported at the time of disease confirmation, while for patients in the 'acute phase' the severity is assessed at the time of semen sampling

^d^The other data in the table are related to 74 (total population) from which a total of 70 semen samples were collected for SARS-CoV-2

^e^Patients with a positive SARS-CoV-2 in the semen sample during acute phase (*n* = 4) were re-tested before discharge (average duration 23 ± 4 days) a SARS‐CoV‐2 was not detected in semen samples

^f^Patients were not vaccinated against COVID-19

^g^10 of 25 patents were vaccinated (6 subjects had been vaccinated twice and 4 subjects had received the vaccine booster). In the pool crude rate, we considered as 75 patients

## Discussion

In our study involving COVID-19 patients, we found no evidence of SARS-CoV-2 in any of the examined semen samples examined, whether during the acute phase or the recovery phase. Throughout the COVID-19 pandemic, numerous studies have attempted to establish the presence of SARS-CoV-2 in the semen of affected individuals. The primary challenges, however, lie in the variations across different protocols employed for sample collection and analysis [4]. The potential for contamination, including environmental, from hands or respiratory droplets, was not eliminated, particularly in home sampling. Only a limited number of studies implemented protocol measures to mitigate contamination in semen samples. Delaroche et al. attempted to analyze the presence of bacterial DNA to evaluate the potential for manual or droplet contamination. In the sole positive sample, a slightly higher concentration of bacterial DNA was observed than in all negative samples. However, the identified bacteria neither confirmed nor ruled out contamination from oropharyngeal secretions during collection [36]. Additionally, the RNA identified in semen may simply be a residue from urinary shedding [43] and many PCR kits commercially available are not designed or validated for semen samples. Finally, many studies did not mention whether the enrolled patients were vaccinated or not and no specific studies on the influence of vaccination on semen sample positivity for SARS-CoV-2 can be found in literature.

Regarding infectivity, only three studies assessed the infectious potential of semen samples collected from individuals with SARS-CoV-2 infection. These samples were cultivated on Vero E6 cells, but researchers did not observe any indications of viral replication. This was observed in both PCR-positive semen samples for SARS-CoV-2 [36] and PCR-negative semen samples for SARS-CoV-2 from patients in the acute stage [39, 42].

It's important to note that if SARS-CoV-2 is detected in semen, it could come from the testis, epididymides, prostate, seminal vesicles and bulbourethral glands [44], although two studies did not find SARS-CoV-2 in expressed prostatic secretion [45, 46]. The testis seems to be a potential site of pathophysiological effects from COVID-19. On one hand, several studies assert the virus's inability to directly affect testicular cells owing to the absence of co-expression of angiotensin-converting enzyme 2 (ACE2) receptor and transmembrane serine protease 2 (TMPRSS2) modulatory protein [10]. From the experimental side animal models have demonstrated the possibility of direct infection of the testis [47]. A recent experimental study demonstrated in vitro that that human spermatozoa are susceptible to SARS-CoV-2 infection, showing a high expression of ACE2 and co-receptors TMPRSS2, Basigin and Cathepsin L. Moreover, authors have found subcellular sites of viral replication by transmission electron microscopy analysis on the ejaculated semen of a COVID-19-affected man (not included in the review for the absence of a PCR test) [48]. Among studies on testicular pathological changes associated with SARS-CoV-2 infection [49], an interesting study focused on the testis of unvaccinated deceased patients, finding COVID-19 in macrophages and spermatogonial cells. Using sensitive nanosensors and specific methodology of RT-qPCR they reliably demonstrated viral detection and activity (subgenomic RNAs) in the testis, while through an in vitro exposure of VERO cells to testicular macerates, they observed viral content in all samples. To note, all 11 included patients experienced severe pulmonary symptoms needing intensive care [50]. However, other studies have failed to demonstrate a direct effect of viral invasion of testicular cells, but rather an effect derived from the exposure to systemic inflammation and/or SARS-CoV-2 antigens [51, 52].

Our study primarily addressed the variations in defining the “acute” and “recovery” phases, considering the different criteria utilized for initial diagnosis, whether symptom- or laboratory-based. The potential long-term persistence of SARS-CoV-2, particularly in young adults without symptoms, poses challenges in accurately discerning individuals with true viral clearance. In a study by Saylam B. et al., where semen samples were collected the day after a positive diagnostic PCR test, a 13.3% (4/30) were positive for SARS‐CoV‐2 in semen was reported. However, with a new PCR test of semen samples approximately 23 ± 4 days after patients recovered, none of the semen samples contained SARS‐CoV‐2 [37]. A notable distinction in our Active Group was the inclusion of patients exclusively with mild or asymptomatic COVID-19 disease, whereas Saylam B. et al. observed a statistically higher SARS‐CoV‐2 detection rate in semen samples of patients with classic COVID‐19 findings on chest computed tomography.

Concerning the secondary outcomes of our study, eight patients (12.2%) in our sample reported genital or sexual symptoms. Compared with a recent systematic review and meta-analysis [11] reporting a 7% occurrence of clinical manifestations such as orchitis or orchiepididymitis, our findings are slightly elevated, possibly due to the inclusion of men with sexual symptoms. Whether these symptoms are directly related to the presence of the virus in genital organs or stem from an indirect inflammatory effect remains uncertain. As shown in Table 3 (Supplemental Information), the percentage of orchitis-like symptoms in studies examining the presence of SARS-CoV-2 in semen samples is not associated with a higher likelihood of detecting the virus in semen. It's noteworthy that many studies reporting positive semen samples did not specify the percentage of patients with or without orchitis-like symptoms.

Although based on a small sample, urinary function (IPSS) and erectile function (IIEF-5) didn’t change from acute to recovered phase in patients with asymptomatic/mild COVID-19 disease and no patients started therapies for LUTS or erectile dysfunction after the SARS-CoV-2 infection.

Finally, although our study doesn’t have a control healthy group, we did not find a significant difference in semen quality between acute and recovered COVID-19 patients. Evidence about the effect of SARS-CoV2on spermatozoa are conflicting: a recent meta-analysis found only partials effects on some sperm functions, not sperm concentration or progressive motility [53], in a short-term period. Another more recent meta-analysis found that SARS-CoV-2 infection may result in decreased sperm concentration only in severe cases [11] and in the acute phase [4]. Probably the presence of only mild/asymptomatic patients in our Acute Group explains of the absence of significant semen quality difference from the Recovered Group.

Limits of our study are the small sample and the absence of a healthy control group. Limited by sample size, we did not perform any subgroup analysis in terms of COVID-19 severity, presence or absence of genital-sexual symptoms during SARS-CoV-2 infection or time of viral clearance. Another limitation in the analysis of sperm quality was the difference in the timing of sexual abstinence between the two groups. In the Active Group, no abstinence limits were applied to collect samples during the early phase of infection, maximizing the likelihood of detecting SARS-CoV-2 in the semen.

## Conclusions

In acute and recovered COVID-19 patients, our study found no SARS-CoV-2 in semen samples. Early reports suggested a low detection rate (1.7%), but caution is needed because of contamination risks and methodological problems. No significantly differences in semen quality were found between acute and recovered COVID-19 patients. Urinary and erectile functions appeared stable across phases.

## Supplementary Information

Supplementary Material 1.

## Data Availability

The datasets generated during and/or analyzed during the current study are available from the corresponding author on reasonable request.

## Abbreviations

ACE2: Angiotensin-Converting Enzyme 2
COVID-19: Coronavirus disease 2019
IPSS: International Prostate Symptom Score
IIEF-5: International Index of Erectile Function 5-items
LUTS: Lower Urinary Tract Symptoms
mRNA: Messenger RNA
RT-PCR: Reverse Transcription-Polymerase Chain Reaction
SARS‑CoV‑2: Severe acute respiratory syndrome coronavirus 2
TMPRSS2: Transmembrane Serine Protease 2
