# Supplementary material for: SARS-CoV-2 in semen: a multicenter prospective study and literature review
Source: Basic Clin Androl. 2024 Dec 2;34:24. doi: 10.1186/s12610-024-00236-z (PMC11610089; doi:10.1186/s12610-024-00236-z)
Supplement: Supplementary file 1 — Supplementary Material 1. [file 12610_2024_236_MOESM1_ESM.docx]

**SARS-CoV-2 in semen: a multicenter prospective study and literature review**

Supplemental Information

**Table 3 – Studies investigating the detection of SARS-CoV-2 in sperm within the medical literature**

| **Studies** | **City, country** | **Study type (quality rating)** | **No. of men** | **Age**  Mean ± SD or median (IQR) or median [range] | **%** **positive nasopharyngeal**  **swab at time^#^ of semen analysis** | **Clinical phase**  **at time of semen analysis**  **(AP, RP)^1^** | **Days from diagnosis^2^**  Mean ± SD or median (IQR) or median [range] | **Severity of COVID-19^3^** | **Detection of SARS‐CoV‐2 in semen samples** | **Control** | **Orchitis-like symptoms** | **Semen parameters** |
| --- | --- | --- | --- | --- | --- | --- | --- | --- | --- | --- | --- | --- |
| Song et al. 2020 [30] | Nanjing, China | Cohort (4) | 12 | 31 [22–38] | 1 (8.3%) | 100% (12/12) RP | 29.4 [14–42] | 8.3% (1/12) A  91.7% (11/12) M | 0% (0) | No | NM | NA |
| Ning et al. 2020 [31] | Wuhan, China | Cohort (4) | 17 | 35 [23–46] | 9 (52.9%) | 100% (17/17) RP | 27 [12–64] | 47.0% (8/17) M  53.0% (9/17) S | 0% (0) | No | 2.7% (3/112) orchidoptosis | NA |
| Pan et al. 2020 [32] | Wuhan, China | Cross-sectional (4) | 34 | 37 [18–55] | NR | 100% (34/34) RP | 31 [8–75] | 100% (34/34) M/Mo | 0% (0) | No | 17.6% (6/34) orchitis-like symptoms | NA |
| Paoli et al. 2020 [33] | Italy | Case report (5) | 1 | 31 | 1 (100%) | 100% (1/1) RP | 8 | 100% (1/1) M | 0% (0) | No | NM | NA |
| Nicastri et al. 2020 [34] | Italy | Case report (5) | 1 | NR | NR | 100% (1/1) AP | NR | 100% (1/1) M | 0% (0) | No | NM | NA |
| Li D et al. 2020 [35] | Shangqiu, China | Cohort (4) | 38 | NR | NR | 39.5% (15/38) AP  60.5% (23/38) RP | NR | NR | 15.8% (6/38) (4 AP, 2 RP) | No | NM | NA |
| Holtmann et al. 2020 [36] | Düsseldorf, Germany | Prospective Cohort control (3b) | 20 | 42.2 ± 9.9 (for RP) | 2 (16.6%) | 100% (2/20) AP  100% (18/20) RP | 45.2 [8-54] (for RP) | 77.8% (14/18) M 22.2% (4/18) Mo | 0% (0) | 0%  (n = 14) | 1 (5.5%) testicular discomfort | Only Mo severity showed an impairment of sperm quality |
| Guo et al. 2021 [37] | Shandong, China | Case Series (4) | 23 | 41.04 ± 11.56 | 0% | 100% (23/23) RP | 32 [26-34] | 78.3% (18/23) M  21.7% (5/23) Mo | 0% (0) | No | NM | 100% (21/21) sperm counts, total motile sperm counts, and sperm morphology were normal |
| Ma et al. 2021 [38] | Wuhan, China | Cross-sectional (4) | 12 | 31.5 [25–46] | 1 (8.3%) | 100% (12/12) RP | 78.5 [56–109] | 8.3% (1/12) M 91.7% (11/12) Mo | 0% (0) | No | NM | 66.7% (8/12) normal semen quality |
| Rawlings et al. 2020 [39] | San Diego, USA | Cross-sectional (4) | 6 | 38 (mean) | 6 (100%) | 100% (6/6) AP | 12 [6–17] | 100% (6/6) M | 0% (0) | No | NM | NA |
| Pavone et al. 2020 [40] | Palermo, Italy | Cross-sectional (4) | 9 | 42 [28–60] | NR | 22.2% (2/9) AP  77.8% (7/9) RP | 39 [7–88] | 11.1% (1/9) A  88.9% (8/9) M | 0% (0) | No | NM | NA |
| Kayaaslan et al. 2020 [41] | Ankara, Turkey | Cross-sectional (4) | 16 | 33.5 [18–54] | 6 (37.5%) | 100% (16/16) AP | 1 (0–7) | 68.8% (11/16) M  31.2% (5/16) Mo | 0% (0) | No | NM | NA |
| Li H et al. 2020 [42] | Wuhan, China | Cross-sectional cohort study (4) | 23 | 40.8 ± 8.5 | 23 (100%) | 100% (23/23) AP | 25.8 (mean) | 60.9% (14/23) M 39.1% (9/23) Mo | 0% (0) | 0%  (n = 22) | NM | 39.1% (9/23) oligozoospermic  60.9% (14/23) significant increase in leucocytes |
| Ruan et al. 2021 [43] | Wuhan, China | Cross-sectional (4) | 70**^4^** | 30.5 [21-49] | 0% (0) | 100% (70/70) RP | NR | 14.9% (11/74) M  41.9% (31/74)  43.2% (32/74) S | 0% (0) | No | 1.35% (1/74) scrotal discomfort (orchitis was ruled out by MRI) | Compared with  healthy-control, sperm concentration, total sperm count and total motility were significantly  declined (n = 55) |
| Temiz et al. 2021 [44] | Istanbul, Turkey | Cross-sectional (4) | 20 | NR | NR | 50% (10/20) AP  50% (10/20) RP | NR | NR | 0% (0) | No | NM | Sperm morphology was significantly lower in the COVID‐19 patients after  treatment vs control (n = 10) |
| Best et al. 2021 [45] | Miami, USA | Prospective Cohort study (3) | 16 | NR | NR | 100% (16/16) RP | NR | NR | 0% (0) | No | 3.4% (1/30) bilateral testis pain suggestive of orchitis | Concentration and total sperm number was significantly lower (n =30) than control (n = 30) |
| Machado et al. 2021 [46] | Arkansas, USA | Cross-sectional study (4) | 15 | 23 [19 – 43] | NR | 100% (15/15) AP | 4 [2 – 8] | 13.3% (2/15) A  86.7% (13/15) M/Mo | 6.6% (1/15) | No | NM | NA |
| Gacci et al. 2021 [47] | Italy | Prospective cross-sectional study (4) | 43 | [30 – 64] | 0 (0%) | 100% (43/43) RP | NR | 12 (27.9%) NH  26 (60.5%) H  5 (11.6%) ICU | 2.3% (1/43) | No | NM | 25.6% (11/43) oligo-crypto-azoospermic |
| Paoli D et al. 2021 [48] | Italy | Prospective cross-sectional (3) | 4 | 58.5 [28 - 61] | 50% (2/4) | 50% (2/4) AP  50% (2/4) RP | 42.5 [17 – 61] | NR | 0 (0%) | 0 | NM | Azoospermia (25%, 1/4) and asthenozoospermia (25%, 1/4) |
| Burke et al. 2021 [49] | Florida, USA | Cross-sectional (4) | 19 | 32 [24-57] | 52.6% (10/19) | 57.9% (11/19) AP  42.1% (8/19) RP | 6 [1 – 28] | 5.3% (1/19) A  10.5% (2/19) M  84.2% (16/19) Mo | 0% (0) | No | NM | NA |
| Gupta et al. 2021 [50] | New Delhi, India | Cross-sectional (4) | 37 | 32.2 ± 5.6 | NA | 100% (37/37) AP | 4.5 ± 0.5 | 64.9% (24/37) M  35.1% (13/37) A | 0% (0) | No | NM | 17/17 normal semen parameters in acute phase |
| Delaroche et al. 2021 [15] | France | Cross-sectional (4) | 32 | 38.8 ± 10.9 | 100% (32/32) | 100% (32/32) AP | 4 [0 - 8] | 16% (5/32) A  84% (27/32) Mo | 3.1% (1/32) | No | 0 (0%) | NA |
| Saylam et al. 2021 [28] | Turkey | Prospective cohort (2b) | 30 | 35.7 ± 6.8 | 100% (30/30) | 100% (30/30) AP | 1 | NR | 13.3% (4/30)**^5^** | No | NM | NA |
| Sharma et al. 2021 [51] | India | Prospective observational study (4) | 11 | 30 [24 – 40] | 0 (%) | 100% (11/11) RP | 44 [19–59] | 81.8% (9/11) M  18.2% ((2/11) Mo | 0% (0) | No | 0 (0%) | NA |
| Fraietta et al. 2022 [18] | Brazil | Prospective cohort (2b) | 22 | 29 [23- 33] | 0% | 100% (22/22) AP | 6 [5-8] | 91.0% (20/22) M  4.5% (1/22) Mo  4.5% (1/22) S | 0% | No | 9.1% (2/22) | No significant difference in seminal parameters at 7, 14 and 21 after the diagnosis (n = 14) |
| Donders et al. 2022 [52] | Belgium | Prospective observational study (3) | 120**^6^** | 34.7 ± 9.1 | NR | 100% (120/120) RP | 52.7 ± 35.1 | 95.8% (115/120) NH  4.2% (5/120) H | 0% (0) | No | NM | 24.6% (29/118) normal  25.4% (30/118) oligozoospermic;  44.1% (52/118) asthenozoospermic  67.0% (79/118) teratozoospermic |
| Pavone C. et al. 2022 [53] | Italy | Cross-sectional (4) | 36 | 41 (mean) | NR | 50% (18/36) AP  50% (18/36) RP | 15.0 [2.0–88.0] | 8.3% (3/36) A  58.3% (21/36) M  33.3% (12/36) S | 0 (0%) | No | NM | NA |
| Edimiris et al. 2023 [17] | Germany | Prospective case-control study (3) | 25**^7^** for three consecutive times | 34.9 (mean) | 25 (100%) | 25 (100%) AP | 4.4 (mean)  17.9 (mean) 81.7 (mean) | 25 (100%) M | 0% (0) | 0% (0)  (n = 12) | 1 (4%) testicular pain | Semen parameter values did not differ significantly between subjects with mild COVID-19 and the control group (n = 12) |
| Present study | Italy | Prospective observational multicentre (3) | 65 | 34.7 ± 11.1 | 23.1% (15/65) | 23.1% (15/65) AP  76.9% (50/65) RP | 197 (185.5 – 204.5) | 24.6% (16/65) A  52.3% (34/65) M  10.8% (7/65) Mo  12.3% (8/65) S | 0 (0%) | No | 12.2% (8/65) genital-sexual symptoms | No significative difference in sperm parameters between active group and recovered group |
| **Studies n = 29** | | | **827** | **33.5**  **Range: 18-64**  **(n= 23)** | **-** | **40.1% (332/827) AP**  **59.8% (495/827) RP** | **-** | **-** | **1.6% (13/827)** | **-** | **-** | **-** |

SD: Standard Deviation; IQR: Interquartile range; AP: Acute Phase; RP: Recovering phase; A: Asymptomatic, M: mild, Mo: moderate, S: severe; H: Hospitalized, NH: Non hospitalized, ICU: Hospitalized with intensive care unit. NM: Not Mentioned; NA: Not Analyzed; ^#^ Last 1-3 days

^1^ Different criteria have been used to define “acute” and “recovery” phase (i.e. two continuous negative SARS-CoV-2 real-time reverse transcriptase-polymerase chain reaction (RT-PCR) assay of pharyngeal swab specimens or substantial resolution on chest CT scans with much lessened symptoms). We report “acute phase” (AP) and “recovery phase” (RP) according to definitions of each study.

^2^ Different criteria have been used to define the initial diagnosis (i.e. day when the symptoms were noticed or first positive pharyngeal swab or using anti-2019-nCoV antibodies).

^3^ For patients in “recovery phase” severity of COVID-19 has been reported at the time of disease confirmation, while for patients in the 'acute phase' the severity is assessed at the time of semen sampling.

^4^ The other data in the table are related to 74 (total population) from which a total of 70 semen samples were collected for SARS-CoV-2.

^5^ Patients with a positive SARS-CoV-2 in the semen sample during acute phase (n = 4) were re-tested before discharge (average duration 23 ± 4 days) a SARS‐CoV‐2 was not detected in semen samples.

^6^ Patients were not vaccinated against COVID-19.

^7^ 10 of 25 patents were vaccinated (6 subjects had been vaccinated twice and 4 subjects had received the vaccine booster). In the pool crude rate, we considered as 75 patients.

LEGEND: This table presents findings from a nonsystematic literature review on the detection rate of SARS-CoV-2 in semen samples from infected patients, aiming to evaluate the overall crude detection rate. The data includes the studies referenced, their locations (city and country), study types with quality ratings, the number and age of male participants, and the percentage of positive nasopharyngeal swabs at the time of semen analysis. It also notes the clinical phase during semen analysis (acute or recovery), the duration from diagnosis, the severity of COVID-19 in participants, and the detection status of SARS-CoV-2 in semen samples. Additionally, the table reports on any control groups, the presence of orchitis-like symptoms, and the semen quality analyzed.
